# Add-On Effect of Postural Instructions to Abdominopelvic Exercise on Urinary Symptoms and Quality of Life in Climacteric Women with Stress Urinary Incontinence. A Pilot Randomized Controlled Trial

**DOI:** 10.3390/ijerph18030928

**Published:** 2021-01-21

**Authors:** Laura Fuentes-Aparicio, Mercè Balasch-Bernat, Laura López-Bueno

**Affiliations:** 1 Department of Physiotherapy, University of Valencia, Gascó Oliag, 5, 46010 Valencia, Spain; laura.fuentes@uv.es (L.F.-A.); laura.lopez@uv.es (L.L.-B.); 2 Physiotherapy in Motion, Multi Speciality Research Group (PTinMOTION), Department of Physiotherapy, University of Valencia, Gascó Oliag, 5, 46010 Valencia, Spain

**Keywords:** abdominal muscles, pelvic floor muscles, postural control, stress urinary incontinence, urinary symptoms, quality of life

## Abstract

The aim of this study was to investigate the add-on effect of postural instructions to an abdominopelvic exercise program on incontinence urinary symptoms (UI symptoms) and quality of life (QoL) in climacteric women with stress urinary incontinence (SUI). A randomized controlled trial was performed with a total of 40 climacteric women with SUI aged between 46 and 75 years old. Participants were randomly assigned to two groups: a group performing an abdominopelvic exercise program (AEP) (*n* = 20) and a group performing abdominopelvic exercise with the addition of postural instructions (AEPPI) (*n* = 20). Primary outcome measures were UI symptoms, UI impact and QoL related to UI (UI-QoL), measured by 48 h Pad Test and International Consultation on Incontinence Questionnaire Short Form (ICIQ-UI-SF), which were assessed at baseline, post-intervention and 3 months follow-up. Secondary outcome was patient’s satisfaction measured by the 100-point Visual Analogic Scale (VAS) only after the intervention. Between-groups differences were observed in terms of UI-QoL immediately after intervention. Within-groups differences were observed between baseline to 3 months follow-up and between post-intervention to 3 months follow-up in AEPPI group (*p* < 0.05) for UI-QoL and UI impact. UI symptoms were improved in both groups between baseline to 3-months follow-up (*p* < 0.05). Patient’s satisfaction was higher in the AEPPI group (*p* < 0.05). The addition of postural instructions to an abdominopelvic exercise program improves UI impact to QoL and patients’ satisfaction in women with SUI.

## 1. Introduction

The climacteric phase is defined as the phase marking the transition from the reproductive to the non-reproductive state (considering the beginning of endocrinological, biological and clinical features of approaching menopause) [1]. According this definition, climacteric includes perimenopause, menopause and postmenopause. Perimenopause period is previous menopause state when the ovaries gradually produces less estrogen and the onset to irregular menstrual cycles. The menopause period is defined retrospectively as the time of the final menstrual period, followed by 12 months of amenorrhea. Postmenopause is described with the period following the final menstrual cycle [2]. During the climacteric period a progressive estrogen loss is produced, which is the most important cause of urogenital atrophy [3]. Vasomotor symptoms occur promptly. Other symptoms such vaginal atrophy, bladder irritations and urinary incontinence (UI) are usually progressive during climacteric transition [4,5]. UI has been defined as the complaint of any involuntary leakage of urine [6]. UI has an average overall prevalence of 20–30%. Its incidence increases with age, with an elevated prevalence in those aged 65 and over, causing loss of autonomy and quality of life (QoL) [7], feelings of distress, loss of self-esteem and social isolation [8]. Moreover, UI leads to an important economic burden [9]. It is estimated that more than 2 million women are affected by some type of UI, with stress urinary incontinence (SUI) being the most common among affected individuals [10,11]. SUI is characterized by involuntary loss of urine without any previous feeling of a need to void, which takes place on the occasion of a physical stress (cough, lifting something heavy, or any other physical activity). SUI can be manifested individually or in combination of urge urinary incontinence (UUI) in variable proportions (mixed urinary incontinence (MUI)) [6].

Pelvic floor muscles training (PFMT) has been recommended for all types of UI [12]. The pelvic floor does not function as an independent entity; its function is also supported by other synergistic muscles [13]. In this regard, the relationship between pelvic floor muscles (PFM), deep erectors and deep abdominal muscles has been confirmed by electromyography (EMG) [14,15]. All these muscles are known as local system cavity [13]. A correct neuromuscular coordination of the trunk muscles would contribute to the maintenance of continence, by controlling intraabdominal and urethral closure pressure.

Current literature supports the use of a global approach in motor control exercise programs, including diaphragm, transversus abdominis (TrA) and PFM training [12]. The study of Fozzatti et al. [16] supports the use of this approach. They demonstrated that global postural reeducation can improve continence by normalizing diaphragmatic function and trunk stability. These muscles may play a key role in the prevention of SUI since a correct diaphragmatic breathing increases the antero-posterior diameter of the abdomen, which is believed to contribute to maintaining the strength and resistance of the abdominal contraction during a sneeze [17].

Many studies have demonstrated the power and specificity of verbal instructions [18,19,20,21,22]. The single use of verbal instructions has been shown to have an impact on the distribution of activity in complementary muscles and postural muscles during different activities without any changes in the exercise performance [19]. More specifically, several authors have studied the effect of verbal instructions on pelvic floor exercises performance, these studies were based in the influence of proprioceptive input in muscle activity timing, coordination, balance and posture [18,21,22]. In the study of Stafford et al. [21], the activation pattern of the PFM in a sample of men was influenced by verbal instructions. Likewise, Vermandel et al. [22] concluded that instructional feedback can improve PFM activation in women who initially were not able to perform a correct PFM contraction in early postdelivery.

However, it is still unknown how verbal instructions regarding posture may influence PFM activation and urinary continence during specific training of abdominopelvic muscles. Therefore, the aim of this study was to investigate the effectiveness of postural instructions added to abdominopelvic exercise program on the improvement of UI symptoms and QoL in women with SUI.

## 2. Materials and Methods

This pilot randomized controlled trial was carried out in the Functional Urology Unit at Dr. Peset University Hospital (Valencia) from 2014 to 2016. The study was approved by the Universitat de València Ethics Committee for Human Research (H1410616852782) and was retrospectively registered at www.clinicaltrials.gov (accessed on 31/10/2018) under the number NCT03727945. The research was conducted in accordance with the ethical principles established by the Declaration of Helsinki and CONSORT guidelines.

Women referred from urology consultations at the hospital were invited to participate in this research. Climacteric women aged between 46–75 years old who had SUI or stress-predominant mixed UI (MUI) were considered for inclusion. The type of UI was diagnosed by experienced urologists, through both urodynamic and clinical assessment, consisting in an exhaustive clinical history and a Q-tip test [23]. Participants were excluded if they had prolapse grade 3 or 4 based on Pelvic Organ Prolapse Quantification Classification (POP-Q) [24], functional impairment (Barthel scale <85 points) [25,26], neurological or cognitive impairment (mini mental examination <24 points) [27], or the presence of any other type of UI. Additionally, women with a score <2 according to the Modified Oxford Grading Scale (0–5) [28] were also excluded. Participation was voluntary, and all participants signed an informed consent prior to commencement of the study. Participants meeting the eligibility criteria were allocated in two groups: abdominopelvic exercise program (AEP) group and abdominopelvic exercise with the addition of postural instructions (AEPPI) group.

### 2.1. Intervention

Both groups performed 12 sessions lasting 40 min with a frequency of once a week [29]. The exercise programs were led by a physical therapist with 10 years of clinical experience in women’s health. In the first session, baseline postural pattern and PFM function were assessed and used as a reference in order to ensure a correct performance of the exercise program in each participant. PFM function was evaluated by both vaginal palpation and the Modified Oxford Grading Scale (0–5) [28]. Then, women were taught how to contract PFM correctly. In the following sessions (2nd to 6th), a progressive specific pelvic floor muscle training was performed. In the 6th session, the participants were instructed on TrA activation, which was then added to the PFMT from 6th to 12th sessions. The degree of difficulty progressed according to different variables, such as the body position (supine decubitus, lateral decubitus, sitting ball, standing and functional tasks), the number of repetitions or the duration of contractions (see Table S1).

All patients performed daily home training exercises during the treatment period and received a document including different abdominopelvic exercises. Adherence to home exercises was controlled by registering times per week, ensuring that all participants accomplished at least 80% of the total home exercise sessions.

In addition, prior to any specific training, participants from AEPPI group were instructed to maintain cervical alignment, scapular relocation and neutral pelvic tilt. These postural adjustments are supported by the biomechanical principles based in a “neutral spine”, where function is maximized and the risk of injury is minimized [30,31]. The physical therapist provided both verbal and manual feedback in order to teach them the correct posture. In order to guide the postural correction, the physiotherapist gently pushed patients’ chin while asking them to maintain cervical alignment. Then, she gently pushed their shoulders backwards to achieve scapular alignment, and their iliacs until the neutral pelvic tilt was reached. Then, participants were asked to practice 3 repetitions of this technique. During the following sessions, the physical therapist continued providing verbal and manual feedback if she deemed it necessary based on visual observation emphasizing the adjustments needed by each participant.

### 2.2. Outcomes

Data from all participants were collected at baseline, immediately after (post-intervention), and 3 months after the intervention (3 months follow-up). Evaluation was performed by the same physiotherapist. All patients completed a standard medical history questionnaire, including sociodemographic and clinical data. The type of UI was assessed according to clinical symptoms and urodynamic assessment.

Primary outcomes were UI symptoms, UI impact and UI-QoL. UI symptoms were quantified through the amount of urine loss, which was measured using a 48 h Pad Test [6]. This test is a standardized method for quantifying urine leakage that can be performed at home [32]. Patients use and replace pads according to their needs over 48 h. Results are calculated by the difference between the net weight of the used pads and the sum of the non-used ones. The score of the Pad Test can be interpreted as mild (4–20 g), moderate (21–74 g) and severe UI (>75 g) [33,34].

UI impact and UI-QoL were measured using the Spanish validation of the International Consultation Incontinence Short-Form (ICIQ-UI-SF) [35]. This questionnaire which was developed by Avery et al. [36], is highly recommended for the evaluation of UI symptoms in women (grade A) [36,37] and it has demonstrated to have a high reliability (α Cronbach = 0.89) [35]. Furthermore, the assessment of QoL has been recommended by the International Continence Society (ICS) as a complement to clinical measures for the evaluation of UI [37].

The ICIQ-UI-SF is made up of 3 questions assessing frequency of the leaks, amount of leakage, and overall impact of UI. This questionnaire also comprises a fourth non-scored item to assess patients’ perception regarding the cause and type of leakage. This item is especially useful in a clinical context but it was not considered in our study. UI impact was obtained from the values of the third question assessing overall impact (“How much does leaking urine interfere with your everyday life?”, ranging from 0 to 10) [38] and UI-QoL was obtained from the final score of the questionnaire (ranging from 0 to 21), which consists in the sum of the 3 questions, with a higher score indicating more severe UI and greater impact on QoL [35,36,37].

Secondary outcome was patient’s satisfaction measured using a 100-point Visual Analogue Scale (VAS) [39,40] after intervention. In our study, higher scores indicate more satisfaction with treatment.

In order to calculate the sample size, an a priori power analysis was conducted using G*Power software (version 3.1.9.2). Assuming an analysis of variance (ANOVA) of repeated measurements, a medium effect size (*d* = 0.5; *η*_p_*^2^* = 0.06), α = 0.05, power = 0.90, and a correlation among repeated measurements of 0.5, a total sample size of 36 participants would be needed to achieve an appropriate power level for this research.

Participants were randomly allocated to two different groups: the first one performed an abdominopelvic exercise program (AEP) (*n* = 20), and a second one in which the subjects underwent the same exercise program with the addition of postural instructions (AEPPI) (*n* = 20). The allocation of the subjects to either the AEP or AEPPI group was based on the output of a random number generator program the software Research Randomizer https://www.randomizer.org (accessed on 15/09/2014) The research team was composed by 3 urologists and a physiotherapist with 20 years of experience. It was not possible to blind the care providers, since it was the physiotherapist who carried out both interventions. Likewise, participants were not blinded as they had been provided with an information brochure explaining the possible interventions. An external assessor was in charge of randomization and was blinded to the concealment of group allocation, as these were numerically coded.

### 2.3. Statistical Analysis

For the statistical analysis, the statistics package SPSS 24.0.0 was used. Data normality was explored using the Shapiro–Wilk test. Descriptive statistics were used to present sociodemographic and clinical data, quantitative variables were described using mean and standard deviation (SD) and number of subjects (*n*) and using frequencies (%) were used for dichotomous variables. Subjects’ characteristics were compared using Student’s *t*- or Chi-square tests. Mixed 2-factor ANOVAs with repeated-measures in the time factor were used to determine significant differences between groups (AEP and AEPPI) and time point (baseline, post-intervention and 3 months follow-up) for the UI symptoms, UI impact and UI-QoL outcomes. Post hoc analysis with the Bonferroni correction was used for the multiple comparison tests. Moreover, a Student’s *t*-test was used for comparing patient’s satisfaction. Effect size was interpreted as small (d = 0.2), medium (d = 0.5) and large (d > 0.8). The significance level was set at 0.05.

## 3. Results

**Figure 1:**
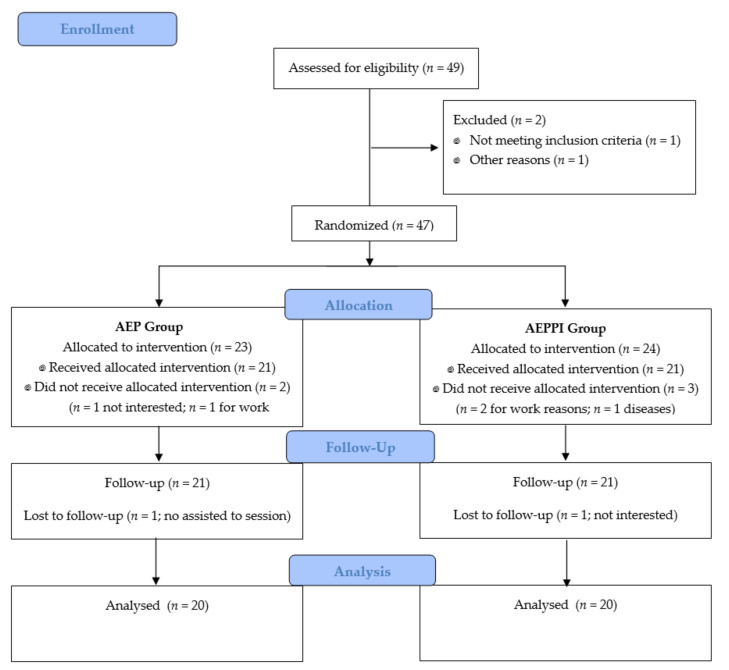
Flow diagram of participants. AEP, Abdominopelvic exercise program; AEPPI, Abdominopelvic exercise program adding postural instructions.

A total of 47 women with SUI were included in the trial and received either an abdominopelvic exercise program (AEP, *n* = 23), or an AEP combined with postural instructions (AEPPI, *n* = 24). Flow diagram depicts the recruitment and retention of participants in this trial (see Figure 1). Three participants in AEP group and 4 in AEPPI group dropped out of the study. The total sample consisted of 40 women aged between 40 and 75 years old, with a mean age of 59.47 (9.34) years. There were no statistically significant differences regarding sociodemographic and clinical variables between two groups (see Table 1). All participants complied with 100% of the exercise program.

### 3.1. Primary Outcomes

ANOVA analysis showed that there were no statistically significant differences between the AEP and AEPPI groups for any variable at baseline (*p* > 0.05) (see Table 2).

Between-group analysis showed significant differences for the UI-QoL measured after the intervention, showing higher improvement for the AEPPI compared to the AEP group (1.29 points).

Within-group analysis showed non-significant differences between baseline and post-intervention for either AEP or AEPPI group. However, when comparing post-intervention and 3 months follow-up, AEPPI group obtained a significant reduction in UI-QoL values (1.75 points), whereas UI symptoms (48 h Pad Test) and UI impact remained unchanged for both groups. Regarding the change between baseline and 3 months follow-up, significant reductions were observed in UI symptoms (48 h Pad Test) for both groups (23.92 g in AEP group and 24.45 g in AEPPI group), while a decrease was observed in UI-QoL values (3.80 points) and UI impact (0.75 points) for the AEPPI group.

### 3.2. Secondary Outcomes

We found significant differences in patients’ satisfaction between groups (*p* = 0.021), in favor of the AEPPI group, who reported higher values (91.5 points) compared to the AEP group (85.5 points).

## 4. Discussion

Several studies have previously investigated the role of posture in continence [33,34]. However, none of them have measured the effect of postural instructions on urinary continence. The present study demonstrated that a 12-session abdominopelvic exercise program supplemented with postural instructions can improve QoL and satisfaction in women with SUI.

Despite slight differences between both groups were observed for UI symptoms (48 h Pad test) and UI impact, these were not statistically significant. The findings of the present study suggest that both UI symptoms and UI impact show a trend to be reduced when postural instructions are added to an abdominopelvic training program. It is important to note that both AEP and AEPPI groups showed an improved rate for UI symptoms (reduction in UI symptoms severity) and UI impact after completion of the intervention. We found a progressive urine leakage reduction in both groups AEP and AEPPI groups (53.80 g ± 78.57 g to 29.88 g ± 51.01 g and 40.70 g ± 44.30 g to 16.25 g ± 24.53 g), respectively, from baseline to 3 months follow-up. Regarding UI impact, although not significant, AEPPI group showed a greater reduction (7.5%) compared to the AEP group (3.7%) from baseline to 3 months follow-up. Similar results were obtained by Hirakawa et al. [41], who compared the effects of PFM training with and without biofeedback in a sample of women with SUI. In this study, the leakage volume measured by the 1 h Pad Test tended to decrease in both groups after 12 weeks of training, but this effect was not significant. Other authors [42] have found a significant improvement in the amount of leakage measured by de 20 min Pad Test when comparing an intervention group with a control group after 12 weeks based on diaphragmatic and abdominopelvic training. In our study, although baseline severity of the UI symptoms did not significantly differ between groups, the two samples were not strictly homogeneous, since participants in the AEP group presented more severe UI symptoms than women in the AEPPI group. We believe that this fact could have influenced the different recovery rate of participants. It may be that the minor improvement experienced by the participants from the AEPPI group has been influenced by the fact that they initially presented less severe UI symptoms, leading to lower chances of improving. Therefore, a longer intervention (more than 3 months) may be necessary for women with less severe symptoms to achieve an important improvement. On the other hand, it could be that women with more severe UI symptoms can benefit more from this program. Regarding UI-QoL and patients’ satisfaction, AEPPI reported greater improvement (reduction of 18% and 91.5% in UI-QoL and patient’s satisfaction scores, respectively) compared to AEP group (reduction of 7% and 85.5% in UI-QoL and patient’s satisfaction scores, respectively) immediately after treatment, suggesting that women with SUI performing abdominopelvic exercise supplemented with postural instructions experienced better QoL and satisfaction compared to those performing the abdominopelvic exercise program without postural instructions. Despite these findings, this study failed to find between-group differences in the mid-term for UI-QoL (3 months follow-up). This result could be considered reasonable, since after the 12 weeks training program no other intervention or home-based exercise was recommended to the participants.

It is necessary to highlight the importance of the findings of the present study since the effect of UI on QoL is more important as UI symptoms, according to previous literature [32,38,43]. No correlation has been observed between objective and subjective assessments in patients with UI. Thus, individual perception is not directly linked to objective measures that quantify the amount of urinary loss.

Furthermore, the number of previous studies that analyzed the impact of postural instructions in abdominopelvic training in patients with UI is limited. Currently, there is a wide variety of abdominopelvic exercise protocols used for the treatment of UI. Different studies have investigated the effect of these interventions on UI impact and QoL, and verbal or tactile feedback concerning the correct posture are frequently provided in their training protocols. To our knowledge, no previous studies evaluated the add-on effect of postural instructions to abdominopelvic training.

Several studies have evaluated the effect of abdominopelvic training in women with UI. Some authors have compared two active groups [16,44,45,46] while others have compared abdominopelvic groups versus a control [42,47]. The studies comparing two active intervention groups reinforce the idea that global abdominopelvic training improves UI symptoms [44,46] and general health perception [45]. Fozzatti et al. [16] evaluated the impact of Global Postural Re-education on SUI symptoms and compared it to PFM training, using another QoL questionnaire, King’s Health Questionnaire (KHQ). In this study, no differences were detected between groups, suggesting that postural global training, even though not including specific exercises for the PFM brings comparable benefits to isolated PFM training in patients’ QoL. Gadheri et al. [46] compared the effect of a group of patients performing stabilization exercise with a group performing the same exercise focusing on PFM. The results of this study differ from our results, showing no differences between both groups regarding QoL, measured by ICIQ-UI-SF. These findings highlight the relevance of different training methods not only focused on PFM. Moreover, Ozengin et al. [45] (detected an increase in general health perception, measured by Prolapse Quality-of-life Questionnaire (P-QoL) [24], in a group performing stabilization exercises compared to PFM training, in women with stage 1 and 2 pelvic organ prolapse. More recently, Ptak et al. [44] revealed that PFM with additional exercise for the TrA muscle was shown to be more effective than isolated PFM exercise in most QoL domains, measured with the International Consultation on Incontinence Modular Questionnaire-Lower Urinary Tract Symptoms quality of life (ICIQ- LUTS) QoL, based on the KHQ. On the other hand, the studies comparing abdominopelvic exercise with a control group suggest that this training method improves the UI symptoms [16,42,48], QoL [47] and muscle function [48]. Hung et al. [42]) reported improvement of both QoL and UI impact in the training group, based on diaphragmatic, deep abdominal and PFM coordinated function, compared to a control group. In contrast to our study, these outcomes were measured by the Symptom Impact Index questionnaire. Also Alves et al. [47] found decreased UI symptoms based on ICIQ-UI-SF in the group of participants performing abdominopelvic exercise. Finally, Tajiri et al. [48] also reported improved UI symptoms following abdominopelvic training as well as an enhanced muscle function, with an increased TrA muscle thickness. Taken together, these results reinforce the idea that global approaches are an appropriate intervention for the treatment of patients presenting UI.

Regarding patient’s satisfaction with the training protocol, the results of our study showed that patients from the AEPPI group were more satisfied, based on VAS, than participants from the AEP group. Previous research [47] has already demonstrated higher values of satisfaction with treatment when comparing abdominopelvic exercise with a control group.

Some studies have demonstrated that patients’ satisfaction is related to adherence to treatment [49]. In particular, it has been stated that perception of the effectiveness of the treatment is considered as a determinant factor contributing to adherence. This issue becomes especially relevant in those treatment programs that require an active role and involvement of the patient to obtain benefits from the therapy.

### Strengths and Limitations

To our knowledge, this study is the first pilot randomized, controlled trial to evaluate the effectiveness of abdominopelvic training supplemented with postural instructions in women with SUI. In addition, in this study the abdominopelvic exercise program, which was supervised by a physical therapist, was reinforced by the performance of home training exercises. The results presented are promising since participants receiving postural instructions achieved better outcomes in QoL and patients’ satisfaction. Moreover, although not significantly, UI symptoms and UI impact were also improved when postural instructions were added to the abdominopelvic program. According to the findings of this study, the implementation of postural instructions regarding spine, scapula and pelvis posture should be highly encouraged amongst physical therapists in the context of abdominopelvic exercise programs performance in their daily clinical practice.

However, this study has several limitations. First, could be a possible limitation in relation to the generalizability of the current study was the small sample size, although the number of recruited participants was sufficient in accordance with an a priori power analysis. Further research with higher sample sizes would help in generalizing the findings of this study. Second, the sample of our study included women with several hormonal status (perimenopausal, menopausal and postmenopausal). In future studies, the effects of postural instructions during abdominopelvic exercise in more homogeneous population in terms of hormonal status could be investigated. Third, the assessment method for UI symptoms in regard to measuring the urinary loss based on pads’ weight (according to the 48 h Pad Test) might also be a study limitation. Third, participants’ adherence to home training exercises was not registered or considered in this study, and this could have biased our results. Despite the findings of this research showing the effectiveness of adding of postural instructions to abdominopelvic exercises in terms of QoL and patients’ satisfaction over time, it would be interesting to investigate the effects of such intervention in the longer term after the completion of the intervention (i.e., 6 months’ and 12 months’ follow up).

According to the results of this study, it is plausible that the addition of exercises more specifically addressed to the PFM activation to the proposed abdominopelvic exercise program could have improved the outcomes related to UI symptoms and UI impact in a sample of women with SUI, when supplementing their exercise program with postural instructions. Besides, further research on how the activation of PFM and abdominopelvic muscles is affected by different instructions is required in order to know whether instructions enhance the influence the activation pattern (EMG activity) of different PFM. This would also allow us to determine whether better outcomes can be achieved with instructions tailored to the women’s continence mechanism. It would also be interesting to find out which postural instructions are more effective for enhancing PFM contraction and UI symptoms in women with SUI. Future studies focusing in the comparison of the effect of different postural instructions on QoL and UI symptoms may help to determine the most appropriate postural approach in this population. Moreover, it could be interesting to investigate the effect of a more extended program, including home-based exercises focused in postural alignment after the 12-week abdominopelvic program proposed in the present study, which may lead to better mid-term outcomes being obtained.

## 5. Conclusions

Both an isolated program of abdominopelvic exercises and a combined program of abdominopelvic exercise supplemented by postural instructions were effective for the improvement of UI-QoL and patients’ satisfaction in a sample of climacteric women with SUI. The abdominopelvic training program with postural instructions was more effective than abdominopelvic training alone in enhancing UI-QoL and patients’ satisfaction immediately after intervention. Moreover, the combined program may have a greater potential for the improvement of UI symptoms and UI impact, but this effect was not demonstrated in this study.

## Figures and Tables

**Table 1:** General sociodemographic and clinical data (*n* = 40).

	AEP (*n* = 20)	AEPPI (*n* = 20)	*p*-Value
Age (years) mean (SD)	61.95 (9.34)	57 (8.88)	0.094 *
BMI kg/m^2^ mean (SD)	27.68 (6.07)	27.70 (6.63)	0.984 *
Menopause age mean (SD)	48.47 (4.87)	48.87 (5.50)	0.850 *
Evolution time-mean (SD)	8.1 (9.49)	8.3 (6.68)	0.939 *
Hysterectomy—*n* (%)	8 (40%)	4 (20%)	0.176 **
Type UI—*n* (%) SUI MUI	14 (70%) 6 (30%)	16 (80%) 4 (20%)	0.478 **
Obstetric history—mean (SD) Parity Cesarean	1.47 (0.009) 0.10 (0.30)	2.00 (1.45) 0.20 (.41)	0.193 * 0.389 *
Hormone replacement—*n* (%)	7 (35%)	4 (20%)	0.300 **
Hormonal status—*n* (%) Perimenopause Menopause Postmenopause	3 (15%) 5 (25%) 12 (60%)	5 (20%) 7 (25%) 8 (35%)	0.235 **
Physical activity—*n* (%) High impact Low impact No activity	3 (15%) 13 (65%) 4 (20%)	4 (20%) 9 (45%) 7 (35%)	0.211 **

Note: * Student’s *t*-test; ** Chi-Square-Statistic; Data are expressed as mean (standard deviation), number of subjects (*n*) and frequency (%); AEP, Abdominopelvic exercise program; AEPPI, Abdominopelvic exercise program adding postural instructions; BMI, body mass index; UI, urinary incontinence; SUI, stress urinary incontinence; MUI, mixed urinary incontinence.

**Table 2:** Results of the comparison for the different variables studied.

	Group	Baseline	Post-Intervention	3m Follow-Up	Comparisons Baseline-Post p ^a^ [95% CI] η_p_^2^	Comparisons Post-3m Follow-Up p ^a^ [95% CI] η_p_^2^	Comparisons Baseline-3m Follow-Up p ^a^ [95% CI] η_p_^2^
***Urinary*** ***symptoms (g)***	**AEP** **AEPPI** p ^c^ [95% CI]; ηp2	53.80(78,57) 40.70(44.30) 0.522 [54.21–28.00]; 0.011	36.13(59.05) 27.43(35.75) 0.579 [40.18–22.79]; 0.008	29.88 (51.01) 16.25(24.53) 0.291 [39.39–12.12]; 0.030	0.084 [37.07–1.73]; 0.467 0.260 [32.17–5.64]; 0.351	0.700 [19.17–6.68]; 0.537 0.097 [23.78–1.41]; 0.557	**0.024 ^b^ [45.31–2.52]; 0.632** **0.017 ^b^ [45.32–3.59]; 0.727**
***UI impact*** ***(0–10)***	**AEP** **AEPPI** p ^c^ [95% CI]; ηp2	2.10(0.875) 2.60(0.882) 0.087 [1.065–0.76]; 0.077	1.84(0.898) 2.20(0.894) 0.220 [0.224–0.940]; 0.040	1.73(0.805) 1.85 (0.745) 0.651 [.390–0.6116]; 0.006	0.597 [0.242–0.768]; 0.27 10.146 [0.892–0.092]; 0.531	1.00 [.552–0.342]; 0.001 0.154 [0.786–0.086]; 0.531	0.83 [.772–0.035]; 0.001 **0.000 ^b^ [1.143–0.357]; 0.962**
***UI-QoL*** ***(0–21)***	**AEP** **AEPPI** p ^c^ [95% CI]; ηp2	1110 (4.21) 13.70(4.00) 0.056 [0.071–5.260]; 0.095	10.36(3.71) 11.65(4.88) **0.042 ^c^ [1.44–4.14]; 0.025**	9.63(3.54) 9.90(3.44) 0.812 [2.538–2.001]; 0.002	1.00 [3.15–1.67]; 0.160 0.210 [4.10–0.603]; 0.610	1.00 [2.69–1.21]; 0.193 **0.031 ^b^ [3.95–0.146]; 0.678**	0.056 [2.97–0.28]; 0.635 **0.000 ^b^ [5.26–2.33]; 1.359**

Note: Data are expressed as mean (Standard deviation); η_p_^2^*,* Partial eta squared; 95% CI, 95% confidence interval; AEP, Abdominopelvic exercise program; AEPPI, Abdo- minopelvic exercise program adding postural instructions; urinary incontinence (UI) symptoms based in 48 h Pad Test registered; UI impact, assessing UI overall impact assessment; UI-QoL, Quality of life related to UI. ^a^ Corresponds to the differences between baseline and post-intervention/post-intervention and 3 months follow-up/baseline and 3 months follow-up; ^b^ Indicates significant difference; ^c^ Corresponds to the differences between AEP and AEPPI. Statistically significant differences *p*-values < 0.05 are highlighted in bold.

## Data Availability

The data presented in this study are available on request from the corresponding author. The data are not publicly available due to privacy or ethical restrictions.

## Supplementary Materials

The following are available online at https://www.mdpi.com/1660-4601/18/3/928/s1, Table S1: Abdominopelvic exercise program.
