# Supplementary material for: Add-On Effect of Postural Instructions to Abdominopelvic Exercise on Urinary Symptoms and Quality of Life in Climacteric Women with Stress Urinary Incontinence. A Pilot Randomized Controlled Trial
Source: Int J Environ Res Public Health. 2021 Jan 21;18(3):928. doi: 10.3390/ijerph18030928 (PMC7908128; doi:10.3390/ijerph18030928)
Supplement: Supplementary file 1 [file ijerph-18-00928-s001.pdf]

Table S1. Abdominopelvic exercise program

**1st session**<br>PFMs proprioception	Patient in crook lying position, with neutral pelvic tilt and the arms by their side.<br>The subject is told to perform an isolated contraction of the PFMs and is instructed to contract the muscles around the vagina "like a drawstring" and to lift them internally.	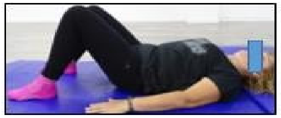
**2nd-3rd session**<br>PFMs static exercise	<u>Fiber I (sub-maximal contraction):</u><br>2 series of 8 repetitions. Each repetition consists of 6 sec holds and 12 sec rest. One min rest between series.<br>1 serie of 8-10 repetitions. Each repetition consists of 7 sec holds and 14 sec rest.<br><u>Fiber II (maximal contraction)</u><br>1 serie of 10 repetitions. Each repetition consists of 2 sec hold and 2 sec rest.<br>1 serie of 10 repetitions of contractions as fast and powerful as possible.	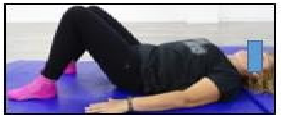
**4th session**<br>PFMs static exercise	<u>Fiber I (sub-maximal contraction)</u><br>2 series of 10 repetitions. Each repetition consists of 7 sec holds and 14 sec rest. One min rest between series.<br>1 serie of 8 repetitions. Each repetition consists of 8 sec holds and 16 sec rest.<br><u>Fiber II (maximal contraction)</u><br>1 series of 10 repetitions. Each repetition consists of 2 sec holds and 2 sec rest.<br>1 serie of 10 repetitions of contractions as fast and powerful as possible.	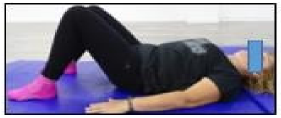
**5th-6th session**<br>PFMs dynamic exercise	<u>Fiber I (sub-maximal contraction):</u><br>1 serie of 10 repetitions. Each repetition consists of 7 sec holds and 14 sec rest. One min rest between series.<br>2 different positions:<br>A. Patient in crook lying position, with neutral pelvic tilt and the arms by their side. The subject performs the bridge exercise and is required to contract the PFMs during the exhalation phase.<br>B. Patient in lateral decubitus with the lower side in 90º flexion and the upper side leg in extension supported on a foam roller.<br><u>Fiber II (maximal contraction):</u><br>2 series of 10 repetitions. Each repetition consists of 2 sec hold and 2 sec rest.<br>1 serie of 10 repetitions of contractions as fast and powerful as possible.<br>The patient performs these contractions only in crook lying position.<br>In the 6th session, the patient is instructed to contract the TrA ("draw in the lower abdominal wall toward the spine"). Then, the subject is told to contract the PFMs followed by the TrA contraction.	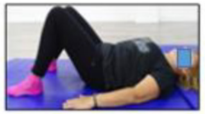 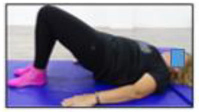 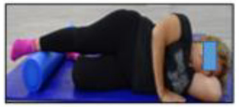
**7th-8th session**<br>PFMs and TrA exercise	The patient in sitting in a gym ball, with neutral pelvic tilt and pushing her fingers in the infraumbilical region.<br>The patient performs the PFMs and TrA contraction as follows:<br><u>Fiber I (sub-maximal contraction):</u><br>2 series of 10 repetitions. Each repetition consists of 6-8 sec holds and 12-16 sec rest. One min rest between series.<br><u>Fiber II (maximal contraction):</u><br>1 serie of 10 repetitions. Each repetition consists of 2 sec holds and 2 sec rest.<br>1 serie of 10 repetitions of contractions as fast and powerful as possible.	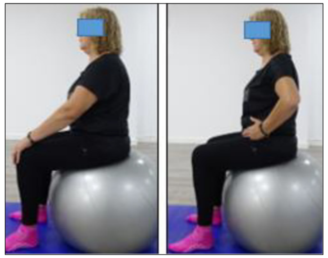
**9th-10th session**	Patient in standing, with neutral pelvic tilt, performs the PFMs and TrA contraction as follows:	

PFMs and TrA exercise	<u>Fiber I (sub-maximal contraction):</u><br>2 series of 10 repetitions. Each repetition consists of 7-9 sec holds and 14-18 sec rest. One min rest between series.<br><u>Fiber II (maximal contraction):</u><br>1 serie of 10 repetitions. Each repetition consists of 2 sec holds and 2 sec rest.<br>1 serie of 10 repetitions of contractions as fast and powerful as possible.<br>These exercises are performed in 2 different positions:<br>A. Standing with slight abduction and external rotation of the hip.<br>B. Standing with slight aduction and internal rotation of the hip.	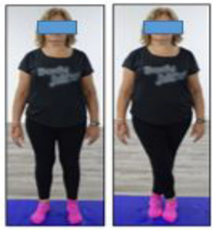
**11th session**<br>PFMs and TrA dynamic exercise	Patient performs the PFMs and TrA contractions as follows:<br>A. The patient in quadruped position extends the lower limb and flexes the contralateral upper limb during the exhalation phase following PFMs and TrA activation. The subject performs 6 repetitions (3 repetitions each side).<br>B. The patient in quadruped position performs anterior and posterior pelvic tilt during the exhalation phase following PFMs and TrA activation. The subject performs 6 repetitions (3 repetitions each pelvic position).<br>C. The patient in sitting in a gym ball, raises one foot while keeping lumbopelvic stability following PFMs and TrA activation. The subject performs 6 repetitions (3 repetitions each side)	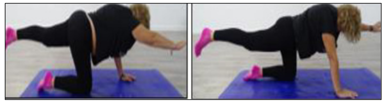<br>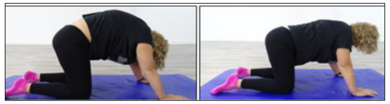<br>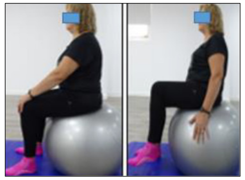
**12th session**<br>PFMs and TrA dynamic exercise	**Description**<br>Patient performs the PFMs and TrA contractions during different functional tasks:<br>A. The patient in sitting in a gym ball performs PFMs and TrA activation while standing up and then picks up the ball from the ground with a squat. Finally, the subject maintains the standing position holding the ball.<br>B. The patient in standing performs PFMs and TrA activation while picking up the ball from the ground with a squat. Then, the patient turns and leaves the ball on the ground.<br>C. The patient in sitting in a gym ball performs PFMs and TrA activation while standing up and sitting down again.<br>Each sequence is repeated 3 times.	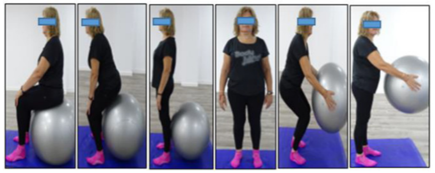<br>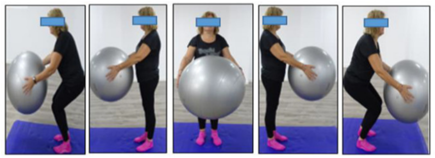<br>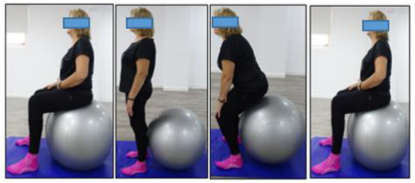

PFMs, pelvic floor muscles; TrA, tranversus abdominis.
